# Identification of Two Structural Elements Important for Ribosome-Dependent GTPase Activity of Elongation Factor 4 (EF4/LepA)

**DOI:** 10.1038/srep08573

**Published:** 2015-02-25

**Authors:** Evelina Ines De Laurentiis, Hans-Joachim Wieden

**Affiliations:** 1 Alberta RNA Research and Training Institute, Department of Chemistry and Biochemistry, University of Lethbridge, Lethbridge, AB, T1K 3M4, Canada

## Abstract

The bacterial translational GTPase EF4/LepA is structurally similar to the canonical elongation factor EF-G. While sharing core structural features with other translational GTPases, the function of EF4 remains unknown. Recent structural data locates the unique C-terminal domain (CTD) of EF4 in proximity to the ribosomal peptidyl transferase center (PTC). To investigate the functional role of EF4's CTD we have constructed three C-terminal truncation variants. These variants are fully functional with respect to binding mant-GTP and mant-GDP as determined by rapid kinetics, as well as their intrinsic multiple turnover GTPase activity. Furthermore, they are able to form stable complexes with the 70S ribosome and 50S/30S ribosomal subunits. However, successive removal of the C-terminus impairs ribosome-dependent multiple turnover GTPase activity of EF4, which for the full-length protein is very similar to EF-G. Our findings suggest that the last 44 C-terminal amino acids of EF4 form a sub-domain within the C-terminal domain that is important for GTP-dependent function on the ribosome. Additionally, we show that efficient nucleotide hydrolysis by EF4 on the ribosome depends on a conserved histidine (His 81), similar to EF-G and EF-Tu.

EF4 (LepA) is a highly conserved 5 domain translational GTPase present in bacteria, as well as the mitochondria and chloroplasts of eukaryotes^1^,^2^,^3^. Previous reports suggest a role of EF4 during retro/back translocation of tRNAs within the ribosome^4^,^5^. The overall structure of EF4 is similar to translation factor EF-G^6^. Domains I, II, III and V are spatially and structurally equivalent to domains I, II, III and V of EF-G (Supplemental Figure S1). However, EF4 lacks a domain corresponding to domain IV of EF-G, which has been shown to be required for EF-G's multiple turnover GTPase activity^7^ and catalysis of translocation^8^. Interestingly, EF4 possesses a unique C-terminal domain (CTD) that spatially occupies a position between domains III and V (Supplemental Figure S1). Both EF4 and EF-G bind to the A site of the ribosome and the available structures of the respective ribosomal complexes reveal that domains I, II, III and V of EF-G and EF4 contact the ribosome in a similar fashion^9^,^10^,^11^. Consistent with its unique structure, the CTD of EF4 interacts differently with the ribosome than domain IV of EF-G. In fact, the majority of contacts made between EF-G's domain IV, P-site tRNA and the bound mRNA are not observed in the respective EF4 - ribosome complex^9^,^11^. Little is known about the detailed function of EF4's CTD, in particular the mechanistic role of its last 44 amino acids, which are not resolved in the structures of the free, as well as the ribosome-bound protein from *Escherichia coli*^6^,^10^. Recent crystallographic data reveal that the CTD of EF4 reaches into the PTC of the ribosome and contacts the acceptor stem of the P-site bound tRNA^11^. Interestingly, this study shows that the C-terminal 44 amino acids of EF4's CTD fold back onto itself and towards EF4's G-domain, hinting at a functional role for the flexibility of these residues.

Although the role of EF4 during back-translocation has been studied in some detail^4^,^5^, the nucleotide-binding and GTP-hydrolysis properties have not been investigated systematically. We have therefore performed a detailed kinetic analysis of the guanine nucleotide-binding properties and GTPase activity of EF4. In order to investigate the catalytic mechanism of GTP hydrolysis and the role of EF4's CTD, we have constructed a GTPase inactive, as well as three sequential C-terminal truncation variants. These variants are compared to the full-length EF4 with respect to their ability to bind guanine nucleotides, associate with the ribosome and to hydrolyze GTP. Our findings suggest that the last 44 C-terminal residues constitute a C-terminal sub-domain that likely has important regulatory roles during EF4's functional cycle.

## Results

### Construction of C-terminal domain truncation variants of EF4

**Figure 1:**
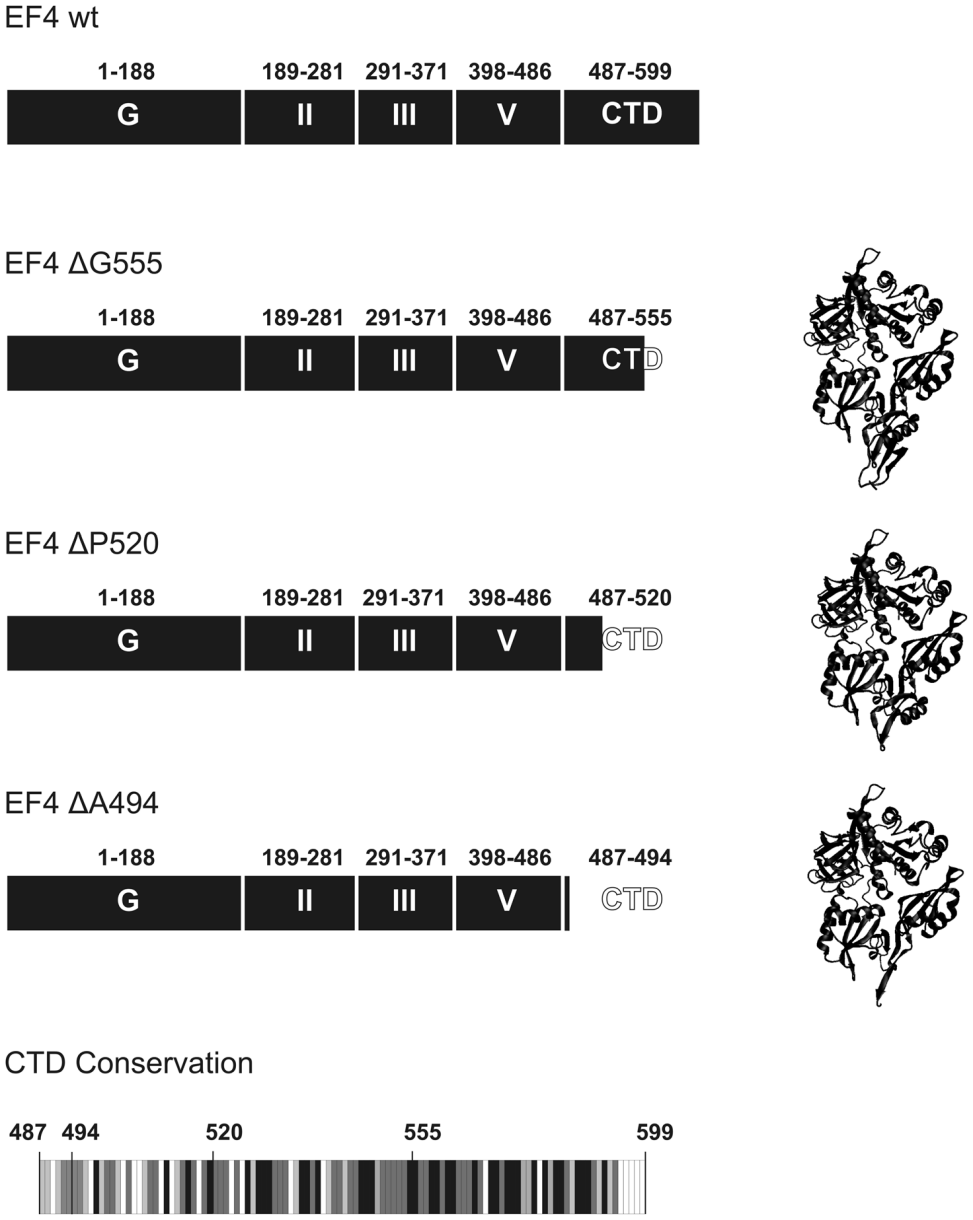
EF4 C-terminal truncation variants. Structural models corresponding to EF4 ΔG555, EF4 ΔP520 and EF4 ΔA494 are represented in cartoon based on the available X-ray crystallographic structure (PDB ID 3CB4^6^). Conservation of the C-terminal domain of EF4 is given as grey scale representation including the position of the three truncations. Black indicates 100%, dark grey 80–100%, light grey 60–80% and white < 60% conservation (Supplemental sequence alignment).

Triggered by the observation that the C-terminal 44 amino acids of EF4's unique CTD were unresolved in previously reported X-ray and cryo-EM structures, we constructed three C-terminal truncated variants of EF4. These variants are based on the published experimentally determined 3-dimensional and predicted secondary structure of EF4^6^,^10^. Interestingly, the recent X-ray crystallographic structure of EF4 from *Thermus thermophilus* bound to the ribosome revealed that the last 44 residues of the CTD can form a mainly helical structural element that folds back onto the protein itself^11^. In agreement with this structure and informed by the original prediction, we truncated the CTD after Gly 555 (ΔG555), removing this positively charged helical element. The additional truncations remove two consecutive β-sheets (ΔP520) directly preceding the C-terminal helical element, as well as a long initial helix (ΔA494) deleting the CTD completely (Figure 1).

### EF4 has a higher affinity for GDP than for GTP

Although significant effort has been made to characterize the function of EF4 *in vivo* and *in vitro*^4^,^12^, the kinetic parameters describing the interaction of EF4 with the respective guanine nucleotides have not been determined. This is particularly surprising as EF4 is a highly conserved GTPase, such as EF-Tu and EF-G, which typically cycle between their active GTP-bound and inactive GDP-bound states as a means of controlling their molecular mechanism of action.

We therefore performed a detailed kinetic analysis of the interaction between purified *E. coli* EF4 and mant-labelled guanine nucleotides using a rapid-kinetics approach. Fluorescence resonance energy transfer (FRET) from two tryptophans located in domain II of EF4 was used to excite a mant-label on the respective guanine nucleotides.

#### Nucleotide dissociation

**Figure 2:**
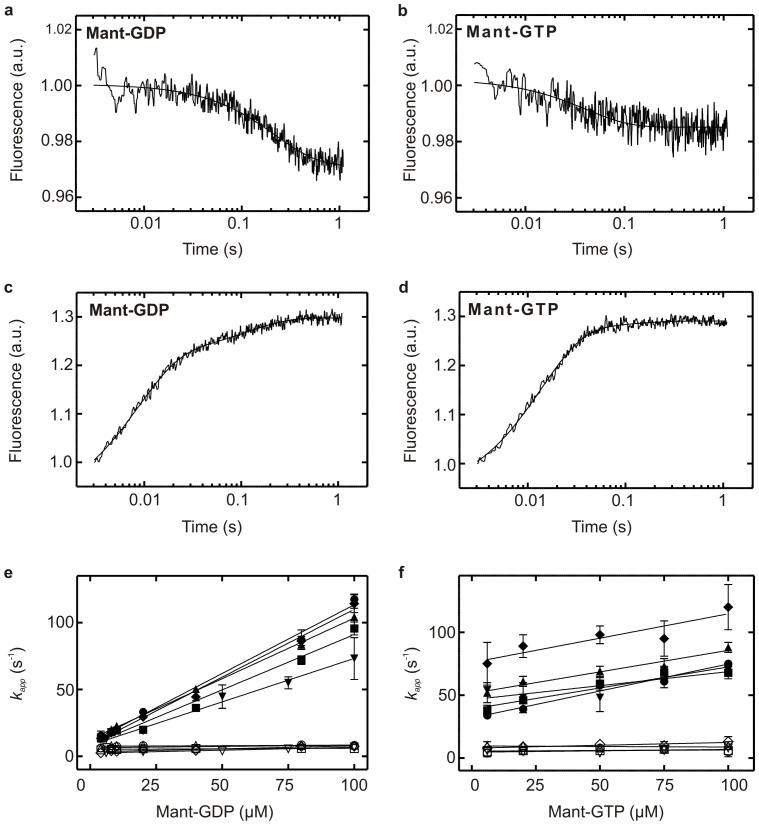
Pre-steady state kinetics of guanine nucleotide binding to EF4. Time courses of (a) mant-GDP (b) mant-GTP dissociation from, and (c) mant-GDP (100 μM) (d) mant-GTP (100 μM) association to EF4 (2 μM) using FRET between intrinsic tryptophan residues in EF4 and the mant-group of the respective nucleotide. Smooth lines represent fits to the obtained fluorescence time courses. Concentration dependence of *k_app_* for (e) mant-GDP and (f) mant-GTP obtained from the respective association time courses. EF4 wild type (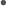
*k_app1_*, 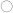
*k_app2_*), EF4 ΔA494 (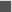
*k_app1_*, 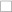
*k_app2_*), EF4 ΔP520 (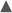
*k_app1_*, 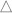
*k_app2_*), EF4 ΔG555 (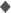
*k_app1_*, 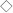
*k_app2_*) and EF4 H81A (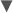
*k_app1_*, 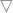
*k_app2_).*

To measure the rate constants of guanine nucleotide dissociation, EF4 was incubated with mant-GDP or mant-GTP and rapidly mixed with a 1000-fold excess of the corresponding non-fluorescent guanine nucleotide. Fluorescence of the mant group excited via FRET was monitored and a rapid decrease in mant fluorescence was observed, representing the dissociation of mant-labelled nucleotide from EF4 (Figure 2a, b). Using this approach, the rate constants (*k_off_*) for mant-GDP and mant-GTP dissociation from EF4 and variants were obtained by fitting the observed fluorescence time courses with a single-exponential function. The respective rate constants are summarized in Table 1. Dissociation of mant-GTP from EF4 is approximately 5-fold faster than for mant-GDP. Removal of the CTD does not affect these rate constants, excluding a potential role of the CTD in nucleotide dissociation.

#### Nucleotide association

The Association rate constants between EF4 and mant-labelled guanine nucleotides were determined by rapidly mixing EF4 with increasing concentrations of the respective mant-labelled guanine nucleotide (Figure 2c and 2d). Time courses of mant-GDP or mant-GTP association to EF4 show biphasic behavior, with only the first rapid phase being dependent on the concentration of the guanine nucleotide. This behavior is consistent with a two-step binding mechanism entailing a conformational change within EF4 following initial binding of the nucleotide. Based on the amplitudes, we observe that the first rapid phase dominates the overall signal (90% of mant-GTP association, 75% of mant-GDP association) (Supplemental Figure S2). This suggests that the large increase in FRET is due to binding of the nucleotide from solution and is followed by a smaller fluorescence change caused by a structural rearrangement.

No differences were observed between wild type EF4 and the respective variants (summarized in Table 1). Furthermore, the obtained values are comparable to the previously determined *k_on_* for mant-GTP and EF-G^13^. The Y-intercepts obtained from the respective *k_on1(GDP/GTP)_* plots (Figure 2e and 2f) are in good agreement with the determined *k_off(GDP/GTP)_* values (Supplemental Table S1) and support a 5-fold faster mant-GTP dissociation rate constant compared to mant-GDP.

#### Equilibrium binding constants

Even though the pre-steady state analysis of nucleotide association revealed a two-step binding process, the corresponding nucleotide dissociation experiments show single-phase kinetics. This suggests that one of the observed dissociation steps is extremely fast and cannot be measured in our system, or that the rate constants for both dissociation steps are of the same magnitude and cannot be discriminated. In order to obtain equilibrium binding constants and to calculate the missing dissociation rate constants, we have used the concentration dependence of the amplitudes (association) to determine the equilibrium binding constants (K_D_) for the respective nucleotides and proteins (Supplemental Figure S2). The determined K_D_ values are summarized in Table 1 and reveal affinities for all studied proteins of approximately 5 μM and 20 μM for mant-GDP and mant-GTP, respectively. From this we were able to determine the second dissociation rate constants (*k_off2_*) using the equation K_D_ = *k_off1_* × *k_off2_*/*k_on1_* × *k_on2_* and observe that they are of similar magnitude as the experimentally observed rate constant (*k_off1_*) (Supplemental Table S1). Therefore, given the small overall amplitude change for the second step, we are not able to discriminate the two consecutive steps in our dissociation experiments.

In summary, our results demonstrate that EF4 has a 5-fold higher affinity for GDP than for GTP, which is different from the observed values for EF-G^13^, but consistent with RF3 that also has an approximately 5-fold higher affinity for GDP than GTP^14^,^15^. Furthermore, the CTD truncation variants bind to mant-labelled guanine nucleotides with a similar affinity as wild type, again excluding a role for the CTD in regulating nucleotide exchange in EF4.

### Truncation of the CTD does not abolish ribosome binding

The recent crystallographic structure of EF4 on the *T. thermophilus* ribosome, together with the observation that the structurally related GTPase BipA requires its unique CTD for ribosome binding, suggests a similar role for EF4's CTD^16^. Alternatively, given that the majority of interactions between EF4 and the ribosome occur through its remaining domains, the CTD may rather play a role in sensing the functional state of the ribosome and therefore might be dispensable for binding. The fact that approximately half of the CTD (residues 545–599, putative C-terminal subdomain) has not been resolved in the available cryo-EM map of EF4 on the *E. coli* ribosome indicates that this region may be able to retain flexibility on the ribosome. To assess whether the CTD and the presence of a P-site tRNA are required for EF4 binding to the ribosome, we have used an ultracentrifugation-based assay to determine EF4-binding to the 70S ribosome and the respective 50S/30S ribosomal subunits.

**Figure 3:**
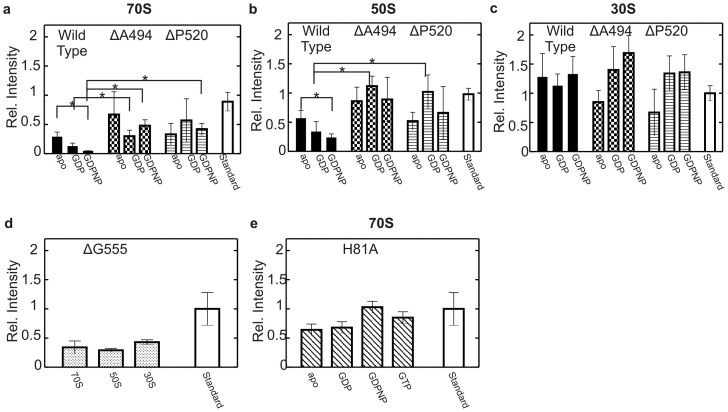
Binding of EF4 and variants to the ribosome in the presence of guanine nucleotides. The intensity of each band following immunodetection was compared to a 20 pmol standard representing stoichiometric binding. Values with a >85% probability to be significantly different are indicated by a star. 2 μM EF4 wild type (filled), ΔA494 (chequered), ΔP520 (striped) (indicated on top) apo or in the presence of various GDP or GDPNP (0.1 mM) (indicated underneath) bound to (a) 0.1 μM 70S ribosome, (b) 0.1 μM 50S ribosomal subunit, (c) 0.1 μM 30S ribosomal subunits. (d) 10 μM EF4 ΔG555 bound to 0.1 μM 70S, 50S or 30S (indicated underneath) in the presence of GDPNP (0.1 mM). (e) 2 μM EF4 H81A bound to 0.1 μM 70S ribosomes in the presence of various guanine nucleotides (GDPNP, GDP, GTP (0.1 mM)) or *apo* (indicated underneath).

In all experiments, EF4 and the respective variants could be detected in the 70S ribosome as well as the 50S/30S ribosomal subunit pellets, indicating that EF4 and all CTD truncation variants are able to associate with the ribosome (Figure 3 and Supplemental Figure S3). Neither EF4 nor the variants were detected in the absence of 70S ribosome or 50S/30S ribosomal subunits (Supplemental Figure S3).

Interestingly, the fraction of EF4 bound to the 70S ribosome as well as the 50S ribosomal subunit (Figure 3a and 3b) was dependent on the nucleotide present during complex formation (fraction of EF4 bound: apo > GDP > GDPNP). No such behavior was seen for 30S ribosomal subunit binding (Figure 3c). Furthermore, nucleotide-dependence of EF4 binding to the 70S or 50S was not observed for the variants (Figure 3a and 3b), suggesting a role for the CTD in nucleotide-dependent regulation of ribosome binding. In comparison, the fraction of EF4 and its variants in the ribosome pellet was larger for the ribosomal subunits (50S < 30S) than for the 70S ribosome and increased with sequential shortening of the C-terminal domain.

Binding of EF4 ΔG555 to the ribosome seemed to be weaker than for the other variants, as a 5-fold higher protein concentration was required to detect EF4 ΔG555 in the ribosome pellet (Figure 3d). This further suggests a role for the putative C-terminal sub-domain during EF4 binding to the ribosome and its 50S and 30S subunits. However, the fact that EF4 ΔG555 was still able to form a complex with the 70S ribosome and the 50S and 30S ribosomal subunits supports the notion that binding of EF4 to the ribosome is achieved mainly through its other domains.

In summary, these experiments demonstrate that the CTD of EF4 is not essential for binding to the ribosome or its subunits and that a P-site bound tRNA is not required.

### Histidine 81 is essential for catalysis in EF4

All translational GTPases interacting with the ribosomal A site contain a conserved histidine residue that is important for efficient GTP hydrolysis on the ribosome^17^,^18^. Aligning the sequence of EF4 with elongation factors EF-Tu and EF-G identifies histidine 81 within *E. coli* EF4 as a candidate for this function. To confirm the required role of histidine 81, we constructed a substitution variant of EF4 containing an alanine at this position (EF4 H81A). Consistent with our assumption, this variant exhibits GTPase activity that is indistinguishable from background hydrolysis levels (data not shown) and that cannot be stimulated by the 70S ribosome (*vide infra*). However, it is still able to bind to the ribosome (Figure 3e). To exclude any effect of the substitution on the nucleotide binding properties, we have determined the association and dissociation rate constants for mant-GDP/GTP and this variant as well as the equilibrium binding constants (K_D_) (Table 1). Our data demonstrate that guanine nucleotide binding is not affected and therefore, like histidine 84 in EF-Tu, histidine 81 is critical for GTP hydrolysis by EF4.

### Truncation of the CTD impairs ribosome-dependent GTPase activity of EF4

**Figure 4:**
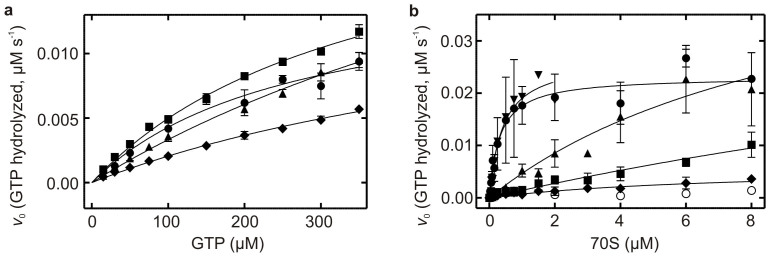
Michaelis-Menten titration of EF4's intrinsic and ribosome-stimulated GTPase activity. (a) Initial rates of GTP hydrolysis are plotted as a function of increasing GTP concentration in the presence of 5 μM EF4 wild type (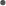), EF4 ΔA494 (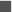), EF4 ΔP520 (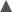), EF4 ΔG555 (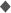). (b) Initial rates of GTP hydrolysis are plotted as a function of increasing 70 S ribosome concentration in the presence of (0.01 μM) EF4 wild type (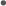), EF-G (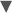), EF4 ΔA494 (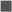), EF4 ΔP520 (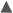), EF4 ΔG555 (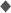), EF4 H81A (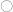).

In order to characterize the intrinsic GTPase activity of EF4 and any putative role the C-terminal domain might play in regulating it, we have performed Michaelis-Menten analyses of EF4 and the three CTD truncation variants reported here (Figure 4a). The determined Michaelis constants (K_M_) and *k_cat_* values are summarized in Table 2. Consistent with a putative role for histidine 81 in the catalysis of GTP hydrolysis (*vide supra*), no hydrolysis of GTP was observed for EF4 H81A and no Michaelis-Menten parameters could be determined for this variant. Interestingly, the K_M_ values obtained for all EF4 CTD truncation variants are in the high micro-molar range (Table 2) and are one order of magnitude larger than the equilibrium binding constants determined here for EF4 and mant-GTP. The obtained *v*_max_ and in turn the determined *k_cat_* for EF4 and respective variants are similar to each other, indicating that the CTD does not modulate its intrinsic GTPase activity.

The GTPase activity of EF4 has previously been shown to be stimulated by the 70S ribosome^4^,^19^. However, no detailed kinetic parameters describing this were reported, preventing a mechanistic interpretation of this interaction. We therefore performed the first detailed Michaelis-Menten analysis of EF4's ribosome-stimulated GTPase activity to investigate a putative role of the CTD on the GTPase activity of EF4 (Figure 4b). K_M_ and *k_cat_* values obtained for full length EF4 are similar to EF-G values (Table 2). Interestingly, the K_M_ values obtained for the EF4 CTD truncation variants are at least one order of magnitude larger than values for the full-length protein. Even though all CTD truncation variants exhibit a similar 10 to 100-fold effect on the K_M_, the *k_cat_* values obtained for the two larger deletions ΔA494 and ΔP520 are within error of those obtained for wild type EF4 and EF-G. However, the *k_cat_* value obtained for the shortest deletion, ΔG555, is 4-fold lower than that of wild type EF4 and the other two truncations (Supplemental Figure S4).

**Figure 5:**
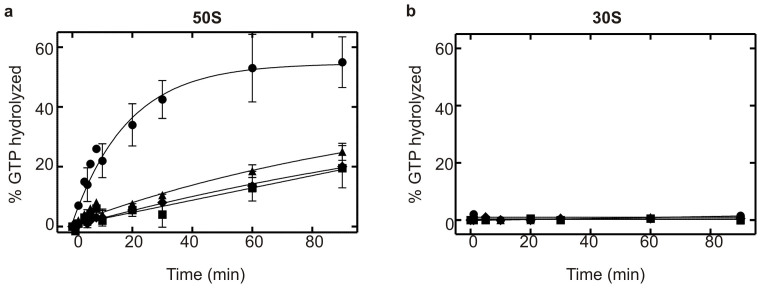
GTPase activity of EF4 in the presence of ribosomal subunits. (a) 50S ribosomal subunit (b) 30S ribosomal subunit stimulated GTPase activity of EF4. The fraction of GTP hydrolyzed is plotted as a function of time. 2 μM EF4 wild type (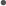), EF4 ΔA494 (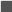), EF4 ΔP520 (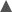) and (10 μM) EF4 ΔG555 (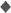) were incubated in the presence of ribosomal subunit (0.1 μM).

Extending our analysis to ribosomal subunits revealed that the 50S ribosomal subunit is able to stimulate the GTPase activity of EF4 and the EF4 CTD truncation variants (Figure 5a), however no stimulation was observed when the 30S ribosomal subunit was present (Figure 5b). We therefore determined Michaelis-Menten parameters for 50S ribosomal subunit stimulation of EF4 and the respective truncation variants (Supplemental Figure S5 and Table S2). We found that the absence of the 30S does not affect the K_M_. However, the *k*_cat_ is reduced 5-fold, which as a consequence reduces the specificity constant. Interestingly, this is similar to the effect that the deletion of the 44 C-terminal amino acids (EF4 ΔG555) has on the 70S stimulated reaction.

### Inhibition of EF4's ribosome-stimulated GTPase activity by Thiostrepton does not require the CTD

**Figure 6:**
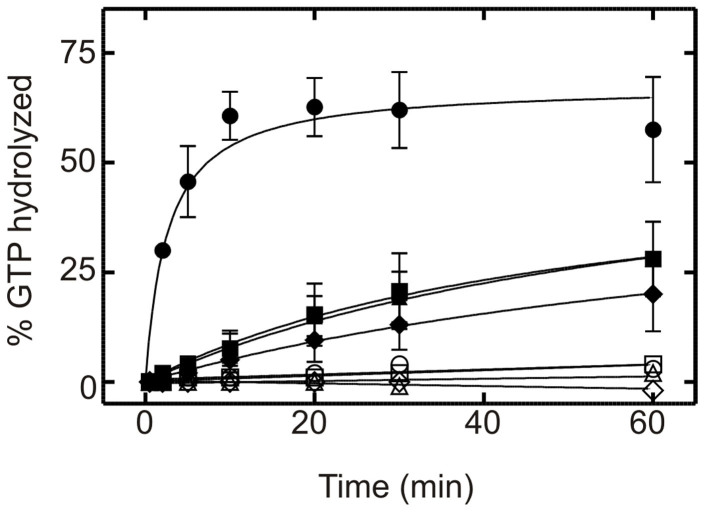
Inhibition of 70S stimulated GTPase activity of EF4 by thiostrepton. GTP hydrolyzed is plotted as a function of time. Samples consisted of 5 μM EF4 wild type (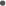), EF4 ΔA494 (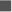), EF4 ΔP520 (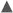) and EF4 ΔG555 (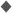) in the presence of 70S (0.1 μM). Open symbols represent the respective GTPase activity in the presence of thiostrepton (10 μM).

Previous studies have shown that thiostrepton efficiently inhibits the ribosome-dependent GTPase activity of EF4^19^. Thiostrepton binds to the GTPase associated center of the ribosome, and likely causes a steric clash with domain V of EF-G or EF4. To investigate whether the unique CTD of EF4 is required for this effect, we have measured time courses of GTP hydrolysis using conditions similar to previous experiments^19^,^20^. In the presence of 10 μM thiostrepton, ribosome-stimulated GTPase activity of the three CTD truncation variants, as well as the full-length EF4, is reduced to background levels (Figure 6). This confirms that thiostrepton inhibits the GTPase activity of EF4 similar to that of EF-G. In addition, the CTD of EF4 does not affect the interaction between domain V of EF4 and the ribosome.

## Discussion

The obtained association and dissociation rate constants governing the interaction between EF4 and mant-GDP/mant-GTP, together with the derived micromolar binding constants (K_D_), reveal that EF4 has a slightly higher affinity for GDP than for GTP (5 μM and 23 μM, respectively). This is different from the translational GTPase EF-G (K_D, GDP_ 17 μM and K_D, GTP_ 7 μM), but is similar to EF-Tu^21^ and RF3^14^,^15^. Given the 10-fold higher concentration of GTP in the cell over GDP^22^, we estimate that during mid-log phase *in vivo*, 66% of free EF4 will be bound to GTP and 33% bound to GDP. This is again different than what is predicted for other translational GTPases, such as EF-G^13^ and RF3^14^,^15^, and suggests that EF4 is likely sensitive to the guanine nucleotide triphosphate-diphosphate ratio and ultimately to the overall energy state of the cell, similar to what has been reported for EttA (energy-dependent translational throttle A)^23^. Such a function would be consistent with the recent published X-ray structure of EF4 bound to the *T. thermophilus* ribosome^11^.

Interestingly, our data indicates that EF4 binding to the 70S ribosome and the 50S ribosomal subunit is nucleotide dependent and that EF4 is capable of binding to the 30S subunit independent of its nucleotide-bound state. The available structural information for EF4 on the ribosome reveals that the last 44 amino acids form a mainly alpha helical structure. Surprisingly, deletion of these 44 amino acids from the CTD seems to reduce binding of EF4 to the ribosome significantly. This truncation follows tyrosine 554 that contacts the 23S rRNA and the CCA-end of the P-site tRNA. However, when the two preceding β-sheets (containing tyrosine 554) are removed, EF4 is no longer inhibited in binding to the ribosome. It seems that in the absence of the C-terminal sub-domain (residues 555 to 599) residues 520 to 555 are able to lock EF4 in a conformation that has a low affinity for the ribosome. Also, removal of residues 520 to 555 of EF4 not only increases the fraction of EF4 bound to the 70S ribosome, as well as the 50S ribosomal subunit, it also abolishes the nucleotide dependence observed for EF4 binding. This demonstrates that the C-terminal 44 amino acids constitute a functionally relevant sub-domain that is likely involved in modulating binding to the ribosome during the still elusive functional cycle of EF4. Such a dynamic behavior of the C-terminal subdomain is supported by the observation that these 44 C-terminal amino acids are connected to the N-terminal part of the CTD by a conserved Gly-Gly linker that could provide conformational flexibility to the C-terminal sub-domain, which in turn would enable it to explore different orientations relative to the rest of the CTD. This is consistent with the observation that in both the X-ray structure of free EF4 and the cryo-EM structure on the ribosome the C-terminal subdomain residues are not resolved^6^,^10^.

While we show that the CTD is not required for intrinsic GTPase activity of EF4, it is important for efficient GTPase activity on the 70S ribosome and the 50S ribosomal subunit. We find that the K_M_ values determined here are at least one order of magnitude higher for the truncation variants than wild type EF4. This is surprising, as our ribosome binding assays indicate that EF4 is stabilized on the ribosome when the CTD is removed. In order to exhibit an increased K_M_ value while having a higher affinity in the binding assays, both the rate of EF4 dissociation from the ribosome and association to the ribosome must be decreased. The fact that the K_M_ values for all the truncation variants are similar indicates that only the last 44 residues of EF4 are sufficient for this effect. It further supports a 44 amino acid C-terminal sub-domain with a key functional role for EF4's interaction with the ribosome, motivating further detailed mechanistic studies.

Consistent with this, the thiostrepton inhibition of EF4's ribosome-stimulated GTPase activity does not require the CTD and further extends the structural similarity between EF-G and EF4 to contacts made between domains I, II, III and V of EF4 and the ribosome, as inhibition is likely caused by interrupting interactions made between domain V of EF4. In addition, our findings indicate that EF4 utilizes a catalytic histidine (His 81) in a structurally equivalent position to histidine 84 in EF-Tu and histidine 92 in EF-G^17^,^24^. Even though EF4 is able to bind to the 30S ribosomal subunit, this is not sufficient to stimulate its GTPase activity. This suggests that ribosome-catalyzed GTP-hydrolysis by EF4 follows a similar mechanism as in EF-G, mainly requiring contributions from the GTPase activating center located on the 50S subunit of the ribosome.

Taken together our findings support a functional role for a C-terminal sub-domain containing the last 44 C-terminal residues of EF4 and suggest that these 44 residues are important for the regulation of EF4's interactions with the ribosome. The whole CTD may act to regulate ribosome interaction of EF4 in a nucleotide dependent manner, and GTPase activity on the ribosome requires the presence of the CTD subdomain. Also, ribosome-stimulated GTPase activity requires a universally conserved histidine residue in position 81, further extending the structural and enzymatic similarities among the translational GTPases.

## Methods

### Reagents

Mant-labelled guanine nucleotides were purchased from Jena Bioscience; guanosine-5′-[γ^32^P]-triphosphate was purchased from Perkin Elmer; thiostrepton, GTP/GDP/GDPNP, antibodies and reagents used for chemiluminescence were purchased from Sigma Aldrich; restriction enzymes were purchased from Fermentas; primers were obtained from Invitrogen; competent cells were from New England Biolabs.

### Cloning and Mutagenesis

The open reading frame *lepA* was PCR amplified from *E. coli* genomic DNA utilizing two primers (5′-AATCATACCATATGAAGAATATACG-3′/5′-CTCCTAAGCTTTATTTGTTGTCTT-3′) and cloned into pET28a using NdeI and HindIII restriction sites (pET28a-*lepA*). Subsequently, C-terminal domain truncation variants (ΔA494, ΔP520, ΔG555) were constructed via PCR using a single reverse primer (5′-TAAGGCTTGCGGCCGCACTCGA-3′) in conjunction with specific forward primers 5′-CGCATCAACACGTTCACCGTTGATTA-3′, 5′-TGGGATCAGATCTTTCATCTTCTCCA-3′, 5′-GCCATAACATTTAGCCAGTACGTTTT-3′ (ΔA494, ΔP520 and ΔG555, respectively). EF4 H81A was constructed through Quickchange™ mutagenesis (Stratagene) employing the primers 5′-TATCGACACCCCAGGCGCCGTAGACTTCTCCTATG-3′ and 5′-CATAGGAGAAGTCTACGGCGCCTGGGGTGTCGATA-3′. Sequence and orientation was confirmed by sequencing (Macrogen).

### Preparation of proteins and ribosomes

Proteins were expressed in *E. coli* BL21-DE3 grown in Luria Bertani medium (10 g tryptone, 5 g yeast extract, 10 g NaCl in 1 L H_2_O, pH 7.5) supplemented with 50 μg/mL kanamycin. For overexpression of EF4 wild type and EF4 H81A, cells were grown at 37°C to mid-log phase (OD_600_ = ~0.6) and induced with 1 mM isopropyl β-D-1-thiogalactopyranoside (IPTG), followed by 3 h at 37°C. EF4 CTD truncation variants were grown (37°C) to mid-log phase (OD_600_ = ~0.6) and induced with 0.5 mM IPTG, followed by 16 h at 16°C. Cells were harvested by centrifugation (5000 × *g* for 10 min at 4°C), flash frozen, and stored at −80°C.

#### EF4 Purification

Cell pellets (approximately 8 g) were resuspended in 7 mL opening buffer (50 mM Tris-Cl pH 8.0 (4°C), 60 mM NH_4_Cl, 7 mM MgCl_2_, 300 mM KCl, 7 mM β-mercaptoethanol, 1 mM phenylmethylsulfonyl fluoride (PMSF), 10 mM imidazole and 15% glycerol) per gram of cells and lysed with 0.1 mg/mL lysozyme followed by centrifugation (30 000 × *g* for 45 min). The obtained EF4 lysates were incubated with 7 mL Ni^2+^-Sepharose (GE Healthcare) in opening buffer, washed with 150 mL opening buffer and 200 mL of wash buffer (opening buffer with 20 mM imidazole). Bound protein was eluted 10 times with 7 mL of elution buffer (opening buffer with 250 mM imidazole) and further purified using size exclusion chromatography (XK26/100 column; Superdex 75 prep grade, GE Healthcare) in buffer A (50 mM Tris-Cl pH 7.5 (4°C), 70 mM NH_4_Cl, 300 mM KCl, 7 mM MgCl_2_ and 15% glycerol). Fractions containing EF4 were pooled, concentrated via ultra-filtration (30 000 MWCO (Sartorius)), flash frozen, and stored at −80°C. The final protein concentration was determined spectrophotometrically (*ε*_280 nm_: 39 935 M^−1^ cm^−1^ for wild type, EF4 H81A and EF4 ΔG555, 38 320 M^−1^ cm^−1^ for EF4 ΔP520 and EF4 ΔA494, calculated using ProtParam^25^) and subsequently confirmed using the Bradford Protein Assay (BioRad).

Vacant ribosomes were purified as previously described^26^ from 50 g of *E. coli* MRE600 cells.

### Fluorescence Stopped-Flow

Rapid-kinetics measurements were performed using stopped-flow apparatus (KinTek, SF-2004) similar to previous studies^20^,^27^. In brief, mant-labelled guanine nucleotides were excited via Fluorescence Resonance Energy Transfer (FRET) from the two tryptophan residues (λ_ex_ = 280 nm) present in domain II of EF4, the resulting fluorescent emission was detected after passing through LG-400-F cut-off filters (NewPort).

#### Dissociation kinetics

200 μM mant-GDP was pre-incubated with 2 μM EF4 in buffer B (50 mM Tris-Cl pH 7.5 (4°C), 70 mM NH_4_Cl, 30 mM KCl and 7 mM MgCl_2_) at 37°C for 15 min. When mant-GTP (200 μM) was used, 2 μM EF4 was pre-incubated in buffer B at 37°C for 15 min with the respective nucleotide in the presence of 3 mM phosphoenolpyruvate (PEP) and 20 U/mL pyruvate kinase (PK). Solutions were rapidly mixed with 2 mM of the corresponding unlabeled guanine nucleotides in buffer B. The resulting fluorescence time courses were best fit with a one-exponential function (Eq. 1)
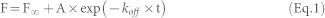
where F is the fluorescence at time t, F_∞_ is the final fluorescence, and *k_off_* is the dissociation rate constant.

#### Association kinetics

The apparent rates for the bimolecular association of mant-nucleotides to EF4 were determined by rapidly mixing 2 μM EF4 with varying concentrations of mant-nucleotides (ranging from 3 to 50 μM after mixing) in buffer B at 20°C. Mant-GTP association experiments were supplemented with 3 mM PEP and 20 U/mL PK. The resulting fluorescence time courses were best fit with a two-exponential function (Eq. 2)
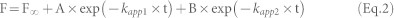
where F is the fluorescence at time t, F_∞_ is the final fluorescence, *k_app1_* and *k_app2_* are apparent rates. The first apparent rate (*k_app1_)* exhibited linear concentration dependence. Based on a bimolecular reaction, the slope obtained by plotting the apparent rates as a function of the nucleotide concentration allows determining the association rate constants (*k_on1(GTP)_* for GTP and *k_on1(GDP)_* for GDP) with a Y-axis intercept corresponding to the rate constant for the reverse reaction (*k_off1_*).

Consistent with the two-step binding mechanism, *k_app2_* did not depend on the nucleotide concentration and was therefore assigned to *k_on2_*. Calculations were performed using TableCurve (Jandel Scientific) and Prism (GraphPad Software).

### Ribosome Binding

Binding of EF4 to the ribosome was assessed using ultracentrifugation. Reactions containing 2 μM EF4 wild type or variants were incubated with either 0.1 μM purified 70S ribosomes or 50S/30S ribosomal subunits in the presence of 0.1 mM of the respective guanine nucleotides (total volume 400 μL) in buffer B for 15 min at 37°C and subsequently loaded onto a 1700 μL 10% sucrose cushion (20 mM Tris-HCl pH 7.6 (4°C), 60 mM NH_4_Cl, 5.25 mM Magnesium acetate, 0.25 mM EDTA, 10% sucrose, 3 mM β-mercaptoethanol) followed by centrifugation at 65 000 × *g* for 18 h. Pellets were resuspended in 40 μL of buffer B and the concentration of 70S/50S/30S in the resuspended pellet was determined at 260 nm using an extinction coefficient of 39103438 M^−1^cm^−1^, 25457162 M^−1^cm^−1^ or 13646276 M^−1^cm^−1^, respectively. Subsequently 20 pmol of ribosomes were analyzed by immunobloting (BioRad Biodot SF). EF4 in the pellet was detected via the His_6_-tag using a monoclonal mouse anti-polyhistidine primary antibody (Sigma) and a peroxidase conjugated secondary goat antibody (Sigma). Chemiluminescence was quantified using a Typhoon 9400 (GE healthcare). Intensities from different experiments were calibrated relative to 20 pmol purified EF4 standards using ImageJ. Significance of each mean (at least 3 replicates) compared to another was assessed using a t-Test (Eq. 3 and 4)
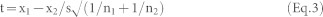
where x_1_ and x_2_ are the calculated means for group 1 and 2, n_1_ and n_2_ are the number of samples in group 1 and 2 and
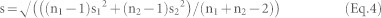
where s_1_ and s_2_ are the standard deviations for group 1 and 2.

Groups that were calculated to have an >85% probability of being different were considered significantly different.

### GTP Hydrolysis

#### Michaelis-Menten Kinetics

To determine the Michaelis-Menten parameters (*k*_cat_ and K_M_) for the intrinsic GTPase activity of EF4, liberation of ^32^P_i_ from [γ^32^P]-GTP was monitored. Guanine nucleotide charging solution (radioactive nucleotide at ~100 dpm/pmol, 3 mM PEP, 20 U/mL PK) was incubated at 37°C for 15 min. Hydrolysis assays were carried out in buffer B. Reaction mixtures contained 5 μM EF4 protein and 0 to 350 μM [γ^32^P]-GTP. Samples were removed at 40 min and quenched in 1 M HClO_4_ with 3 mM potassium phosphate and extracted using 20 mM Na_2_MoO_4_ and isopropyl acetate. The amount of ^32^P_i_ liberated was quantified by scintillation counting (Perkin-Elmer Tri-Carb 2800TR). Background radioactivity was subtracted and the concentration of GTP hydrolyzed as a function of time was calculated and plotted against increasing GTP concentration.

For the ribosome-stimulated GTP hydrolysis of EF4 the reaction contained 0.01 μM protein, 100 μM [γ^32^P]-GTP and 0 to 8 μM 70S or 0 to 4 μM 50S. Samples were removed at various time points (10 to 40 min), quenched, ^32^P_i_ extracted, and quantified as above. Initial rates were calculated and plotted against increasing 70S/50S concentration. Data for both intrinsic and ribosome-stimulated GTP hydrolysis was analyzed using Eq. 5.
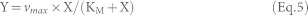


#### Ribosome-stimulated GTPase activity of EF4

Hydrolysis assays were carried out in buffer B and guanine nucleotide charging solution was incubated as described above. Reaction mixtures for 50S and 30S reactions contained 2 μM EF4, 100 μM [γ^32^P]-GTP and 0.1 μM 50S or 30S. Reaction mixtures containing 70S ribosomes and thiostrepton contained 5 μM protein, 100 μM [γ^32^P]-GTP and 0.1 μM 70S (+10 μM thiostrepton and ~0.3% Dimethyl sulfoxide when present). Samples were removed at various time points (0 to 90 min) and analyzed as described above. Background radioactivity from ribosomes was subtracted and the percentage of GTP hydrolyzed was plotted as a function of time.

## Author Contributions

E.D. and H.J.W. designed the research; E.D. carried out all experiments; E.D. and H.J.W. wrote the manuscript. All authors reviewed the manuscript.

## Supplementary Material

Supplementary Information Supplementary Data

## Figures and Tables

**Table 1:** Summary of mant-guanine nucleotide kinetics and affinities to EF4 and variants (20°C)

	*k_off (GDP)_* (s^−1^)	*k_off (GTP)_* (s^−1^)	*k_on1 (GDP)_* (μM^−1^s^−1^)	*k_on1 (GTP)_* (μM^−1^s^−1^)	*k_on2 (GDP)_* (μM^−1^s^−1^)	*k_on2 (GTP)_* (μM^−1^s^−1^)	K_D_ Mant-GDP (μM)	K_D_ Mant-GTP (μM)
Wild Type	4.2 ± 0.3	21 ± 4	1.0 ± 0.1	0.4 ± 0.1	6.4 ± 0.6	5.5 ± 1.4	5 ± 1	23 ± 11
*Δ*A494	2.9 ± 0.5	28 ± 5	0.8 ± 0.1	0.3 ± 0.1	5.1 ± 0.2	5.4 ± 0.5	3 ± 1	18 ± 5
*Δ*P520	3.4 ± 0.1	34 ± 12	0.9 ± 0.1	0.3 ± 0.1	7.4 ± 0.4	9.6 ± 1.0	5 ± 1	24 ± 5
*Δ*G555	4.9 ± 0.9	14 ± 4	1.0 ± 0.1	0.4 ± 0.1	3.3 ± 0.4	7.9 ± 0.8	4 ± 1	32 ± 11
H81A	2.8 ± 0.3	29 ± 1	0.7 ± 0.1	0.2 ± 0.1	2.3 ± 0.3	4.7 ± 1.1	6 ± 1	16 ± 9
EF-G^a^	300	7	n.d.	0.6 ± 0.1	n.d.	n.d.	17	7

a) 13.

**Table 2:** Intrinsic and 70S ribosome-stimulated GTPase activity of EF4 and variants

	K_M_ (μM)		*k_cat_* (s^−1^)		*k_cat_*/K_M_ (s^−1^M^−1^)	
	Intrinsic	70 S	Intrinsic	70 S	Intrinsic	70 S
EF4	270 ± 90	0.32 ± 0.08	0.003 ± 0.001	2.3 ± 0.3	11 ± 5	7 ± 2
*Δ*A494	370 ± 35	36 ± 26	0.005 ± 0.001	5 ± 3	14 ± 3	0.14 ± 0.13
*Δ*P520	900 ± 200	10 ± 5	0.007 ± 0.001	5 ± 2	8 ± 2	0.5 ± 0.3
*Δ*G555	700 ± 100	7 ± 3	0.003 ± 0.001	0.6 ± 0.2	4 ± 2	0.09 ± 0.05
EF-G	n.d.	0.40 ± 0.10	n.d.	2.7 ± 0.3	n.d.	7 ± 2

## References

[b1] Bauerschmitt H., Funes S. & Herrmann J. M. The membrane-bound GTPase Guf1 promotes mitochondrial protein synthesis under suboptimal conditions. J. Biol. Chem. 283, 17139–17146, 10.1074/jbc.M710037200 (2008). 18442968

[b2] Ji D. L., Lin H., Chi W. & Zhang L. X. CpLEPA is critical for chloroplast protein synthesis under suboptimal conditions in *Arabidopsis thaliana*. Plos One 7 10.1371/journal.pone.0049746 (2012). PMC3499520 23166764

[b3] Margus T., Remm M. & Tenson T. Phylogenetic distribution of translational GTPases in bacteria. BMC Genomics 8, 10.1186/1471-2164-8-15 (2007). PMC1780047 17214893

[b4] Qin Y. *et al.* The highly conserved LepA is a ribosomal elongation factor that back-translocates the ribosome. Cell 127, 721–733, 10.1016/j.cell.2006.09.037 (2006). 17110332

[b5] Pech M. *et al.* Elongation factor 4 (EF4/LepA) accelerates protein synthesis at increased Mg^2+^ concentrations. Proc. Natl. Acad. Sci. USA 108, 3199–3203, 10.1073/pnas.1012994108 (2011). 21300907 PMC3044372

[b6] Evans R. N., Blaha G., Bailey S. & Steitz T. A. The structure of LepA, the ribosomal back translocase. Proc. Natl. Acad. Sci. USA 105, 4673–4678, 10.1073/pnas.0801308105 (2008). 18362332 PMC2290774

[b7] Savelsbergh A., Matassova N. B., Rodnina M. V. & Wintermeyer W. Role of domains 4 and 5 in elongation factor G functions on the ribosome. J. Mol. Biol. 300, 951–961, 10.1006/jmbi.2000.3886 (2000). 10891280

[b8] Martemyanov K. A. & Gudkov A. T. Domain IV of elongation factor G from *Thermus thermophilus* is strictly required for translocation. FEBS Letters 452, 155–159 (1999). 10386581 10.1016/s0014-5793(99)00635-3

[b9] Gao Y. G. *et al.* The structure of the ribosome with elongation factor G trapped in the posttranslocational state. Science 326, 694–699, 10.1126/science.1179709 (2009). 19833919 PMC3763468

[b10] Connell S. R. *et al.* A new tRNA intermediate revealed on the ribosome during EF4-mediated back-translocation. Nat. Struct. Mol. Biol. 15, 910–915, 10.1038/nsmb.1469 (2008). 19172743

[b11] Gagnon M. G., Lin J., Bulkley D. & Steitz T. A. Crystal structure of elongation factor 4 bound to a clockwise ratcheted ribosome. Science 345, 684–687, 10.1126/science.1253525 (2014). 25104389 PMC9153294

[b12] March P. E. & Inouye M. GTP-binding membrane protein of *Escherichia coli* with sequence homology to initiation factor 2 and elongation factors Tu and G. *Proc*. Natl. Acad. Sci. USA 82, 7500–7504 (1985). 10.1073/pnas.82.22.7500 PMC390844 2999765

[b13] Wilden B., Savelsbergh A., Rodnina M. V. & Wintermeyer W. Role and timing of GTP binding and hydrolysis during EF-G-dependent tRNA translocation on the ribosome. Proc. Natl. Acad. Sci. USA 103, 13670–13675, 10.1073/pnas.0606099103 (2006). 16940356 PMC1564220

[b14] Peske F., Kuhlenkoetter S., Rodnina M. V. & Wintermeyer W. Timing of GTP binding and hydrolysis by translation termination factor RF3. Nucleic Acids Res. 42, 1812–1820, 10.1093/nar/gkt1095 (2014). 24214994 PMC3919579

[b15] Koutmou K. S., McDonald M. E., Brunelle J. L. & Green R. RF3:GTP promotes rapid dissociation of the class 1 termination factor. RNA 30, 609–620, 10.1261/rna.042523.113 (2014). 24667215 PMC3988563

[b16] deLivron M. A., Makanji H. S., Lane M. C. & Robinson V. L. A novel domain in translational GTPase BipA mediates interaction with the 70S ribosome and influences GTP hydrolysis. Biochemistry 48, 10533–10541, 10.1021/bi901026z (2009). 19803466

[b17] Daviter T., Wieden H. J. & Rodnina M. V. Essential role of histidine 84 in elongation factor Tu for the chemical step of GTP hydrolysis on the ribosome. J. Mol. Biol. 332, 689–699 (2003). 12963376 10.1016/s0022-2836(03)00947-1

[b18] Chen Y., Feng S., Kumar V., Ero R. & Gao Y.-G. Structure of EF-G-ribosome complex in a pretranslocation state. Nat. Struct. Mol. Biol. 9, 1077–1084, 10.1038/nsmb.2645 (2013). 23912278

[b19] Walter J. D., Hunter M., Cobb M., Traeger G. & Spiegel P. C. Thiostrepton inhibits stable 70S ribosome binding and ribosome-dependent GTPase activation of elongation factor G and elongation factor 4. Nucleic Acids Res. 40, 360–370, 10.1093/nar/gkr623 (2012). 21908407 PMC3245911

[b20] Shields M. J., Fischer J. J. & Wieden H. J. Toward understanding the function of the universally conserved GTPase HflX from *Escherichia coli*: a kinetic approach. Biochemistry 48, 10793–10802, 10.1021/bi901074h (2009). 19824612

[b21] Gromadski K. B., Wieden H. J. & Rodnina M. V. Kinetic mechanism of elongation factor Ts-catalyzed nucleotide exchange in elongation factor Tu. Biochemistry 41, 162–169 (2002). 11772013 10.1021/bi015712w

[b22] Buckstein M. H., He J. & Rubin H. Characterization of nucleotide pools as a function of physiological state in *Escherichia coli*. J. Bacteriol. 190, 718–726, 10.1128/JB.01020-07 (2008). 17965154 PMC2223692

[b23] Boel G. *et al.* The ABC-F protein EttA gates ribosome entry into the translation elongation cycle. Nat. Struct. Mol. Biol. 21, 143–151, 10.1038/nsmb.2740 (2014). 24389466 PMC4101993

[b24] Pulk A. & Cate J. H. Control of ribosomal subunit rotation by elongation factor G. Science 340, 1235970, 10.1126/science.1235970 (2013). 23812721 PMC4274944

[b25] Wilkins M. *et al.* Protein identification and analysis tools in the ExPASy server. 2-D Proteome Analysis Protocols 112, 531–552, 10.1385/1-59259-584-7,531 (1999). 10027275

[b26] Milon P. *et al.* Transient kinetics, fluorescence, and FRET in studies of initiation of translation in bacteria. Methods Enzymol. 430, 1–30 (2007) 17913632 10.1016/S0076-6879(07)30001-3

[b27] De Laurentiis E. I., Mo F. & Wieden H. J. Construction of a fully active Cys-less elongation factor Tu: Functional role of conserved cysteine 81. BBA-Proteins Proteom. 1814, 684–692, 10.1016/j.bbapap.2011.02.007 (2011). 21338717

