# Supplementary material for: Identification of Two Structural Elements Important for Ribosome-Dependent GTPase Activity of Elongation Factor 4 (EF4/LepA)
Source: Sci Rep. 2015 Feb 25;5:8573. doi: 10.1038/srep08573 (PMC4339808; doi:10.1038/srep08573)
Supplement: Supplementary Information — Supplementary Data [file srep08573-s1.pdf]

Supplemental Data for:

Identification of Two Structural Elements Important for Ribosome-Dependent GTPase Activity of Elongation Factor 4 (EF4/LepA).

Evelina Ines De Laurentiis[1,2] and Hans-Joachim Wieden[1,3]

[1]Alberta RNA Research and Training Institute, Department of Chemistry and Biochemistry, University of Lethbridge, Lethbridge, AB, T1K 3M4, Canada

[2] Current address: Max-Planck Institute for Biophysical Chemistry, Department of Cellular Biochemistry, Am Fassberg 11, 37077 Göttingen, Germany

[3] To whom correspondence should be addressed. Tel: 001 403 329 2303; Fax: 001 403 329 2057; Email: hj.wieden@uleth.ca

Supplemental Figures

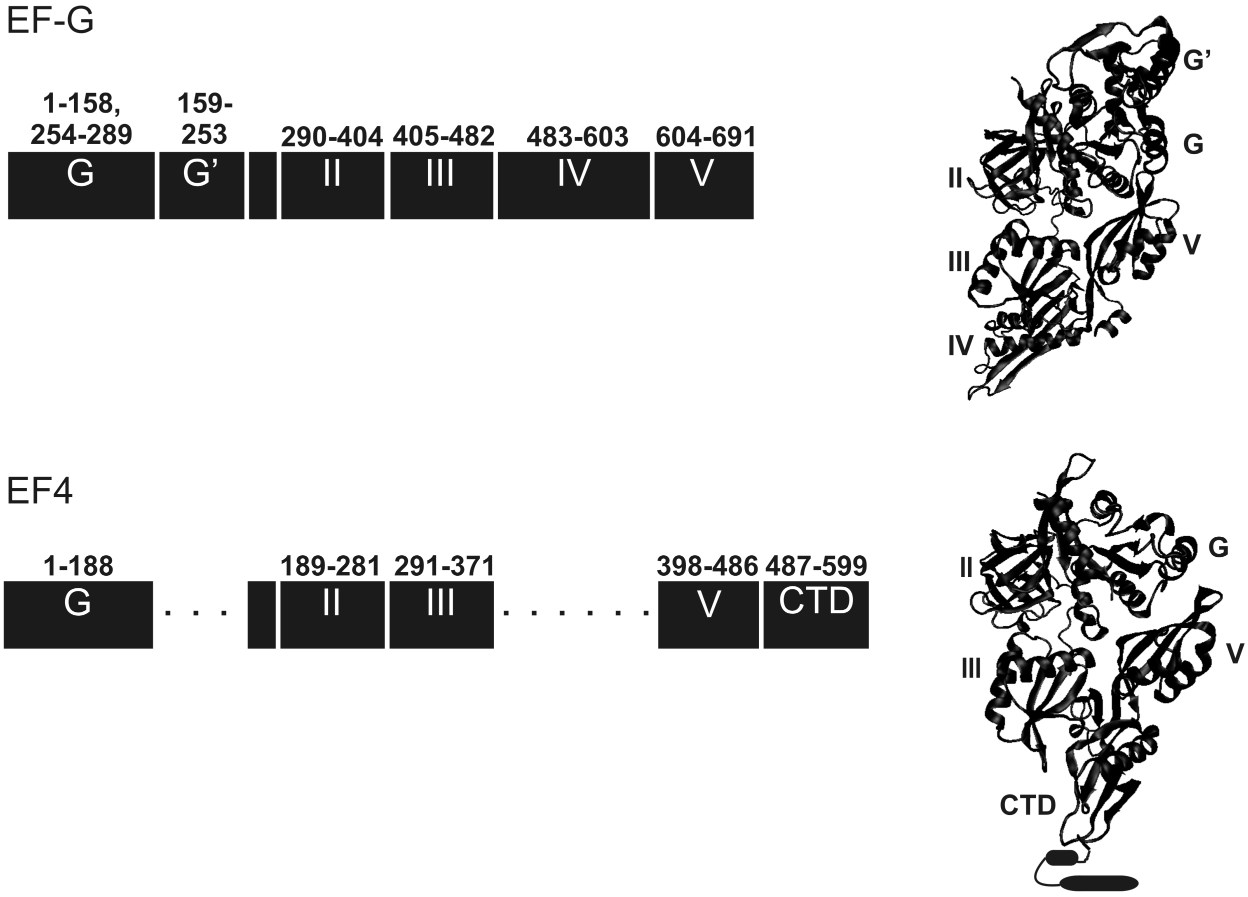

Figure S1: Structural comparison of EF4 and EF-G. The domain arrangement is represented on the left, the corresponding residues within the domain are indicated above the respective domain. Cartoon representations of EF-G from *Thermus thermophilus* (PDB ID 1FNM [1]) and EF4 from *Escherichia coli* (PDB ID 3CB4 [2]) are shown on the right. Domains are labelled as domain I (G-domain and G'-domain), domain II, domain III, domain IV, domain V and the CTD. The previously unresolved and likely flexible portion of the CTD (residues 556 to 599) is represented as a helix turn helix cartoon based on the recent X-ray crystallographic structure[3].

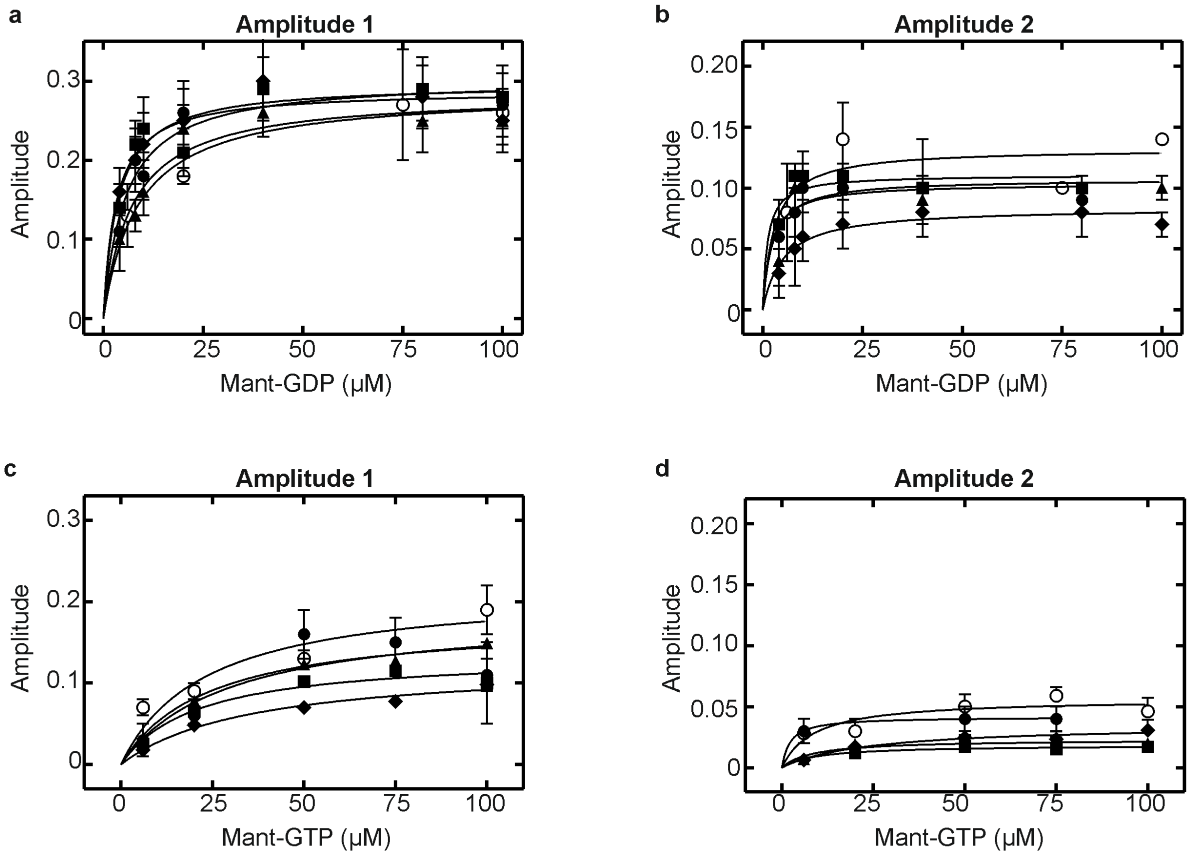

Figure S2: Amplitude of fluorescence change upon binding of mant-nucleotides to EF4. EF4 wild type (●), EF4 ΔA494 (■), EF4 ΔP520 (▲), EF4 ΔG555 (◆) and EF4 H81A (○). Concentration dependence of amplitude 1 (a) and amplitude 2 (b) obtained from fluorescence time courses for mant-GTP and amplitude 1 (c) and amplitude 2 (d) obtained for mant-GDP association.

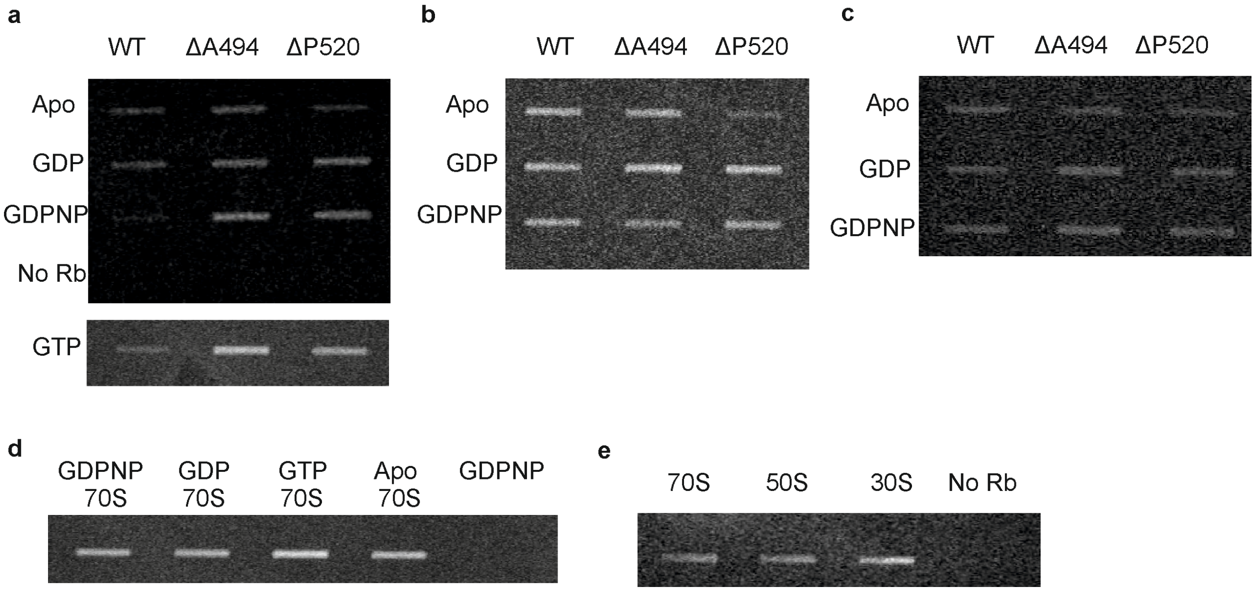

Figure S3: **Immunodetection of EF4 bound to the ribosome following ultracentrifugation.** (a) EF4 proteins (2 μM) (indicated on top) bound to the 70S (0.1 μM) ribosome in the presence of various guanine nucleotides (GTP, GDPNP, GDP (0.1 mM)) or *apo* indicated on the left. Pellet following ultracentrifugation of EF4 proteins bound to GDPNP in the absence of ribosome is shown (No Rb). (b) EF4 proteins (2 μM) bound to the 50S (0.1 μM) subunit in the presence of various guanine nucleotides (GDPNP, GDP (0.1 mM)) or *apo*. (c) EF4 proteins (2 μM) bound to the 30S (0.1 μM) subunit in the presence of various guanine nucleotides (GDPNP, GDP (0.1 mM)) or *apo*. (d) EF4 H81A (2 μM) bound to the 70S (0.1 μM) in the presence of various guanine nucleotides (GDPNP, GDP, GTP (0.1 mM)) or *apo* indicated on top. (e) EF4 ΔG555 protein (10 μM) bound to the 70S, 50S and 30S (0.1 μM) in the presence of GDPNP (0.1 mM).

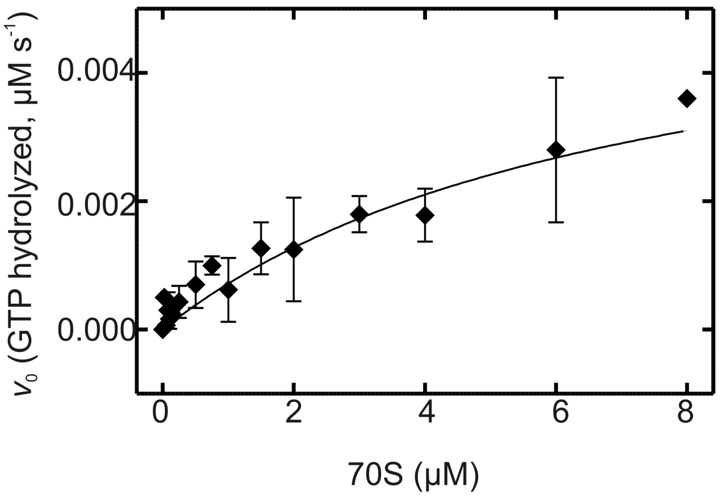

Figure S4: Michaelis-Menten titration of 70S ribosome stimulated GTPase activity of EF4 ΔG555. Initial rates of GTP hydrolysis are plotted as a function of increasing 70S ribosome concentration in the presence of (0.01 µM) EF4 ΔG555.

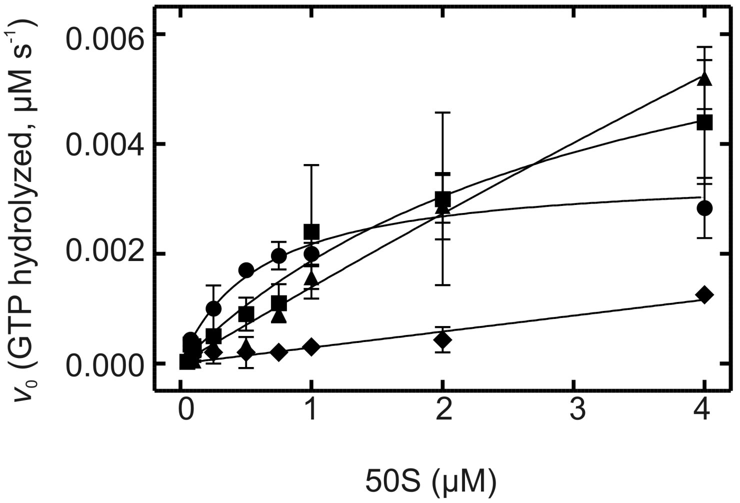

Figure S5: Michaelis-Menten titration of 50S ribosome subunit stimulated GTPase activity of EF4. Initial rates of GTP hydrolysis are plotted as a function of increasing 50S ribosome concentration in the presence of (0.01 µM) EF4 wild type (●), EF4 ΔA494 (■), EF4 ΔP520 (▲), EF4 ΔG555 (◆).

Supplemental Tables

Table S1: Summary of dissociation rate constants determined for mant-GDP and mant-GTP from EF4.

	EF4	ΔA494	ΔP520	ΔG555	H81A
$k_{off1(GDP)}$ y-int (s^{-1})	11 ± 2	8 ± 3	13 ± 1	9 ± 2	8 ± 2
$k_{off1(GTP)}$ y-int (s^{-1})	32 ± 3	39 ± 3	51 ± 3	76 ± 6	46 ± 6
$k_{off2(GDP)}$ (s^{-1})	8 ± 2	4 ± 2	10 ± 2	3 ± 2	4 ± 2
$k_{off2(GTP)}$ (s^{-1})	2.4 ± 1.5	1.0 ± 0.5	1.4 ± 0.6	7.2 ± 3.8	0.5 ± 0.4

Table S2: 50S ribosome dependent GTPase activity of EF4 and three truncation variants.

Protein	K_M (μM)	v_{max} (μM s^{-1})	k_{cat} (s^{-1})	k_{cat}/K_M (s^{-1} $μM^{-1}$)
EF4	0.6 ± 0.1	0.0035 ± 0.0003	0.4 ± 0.1	0.7 ± 0.2
ΔA494	3 ± 2	0.008 ± 0.003	0.8 ± 0.3	0.3 ± 0.2
ΔP520	>1	>0.006	>0.6	NA
ΔG555	>1	>0.001	>0.1	NA

Sequence Alignment

Sequences (SwissProt) and Organisms used in the EF4/LepA multiple sequence alignment

Accession Number	Organism	Seq. identity to *E. coli* (%)
P60785	*Escherichia coli*	
Q9X1V8	*Thermotoga maritima*	50
Q72KV2	*Thermus thermophilus*	55
A0RIT7	*Bacillus thuringiensis*	56
C3P8M5	*Bacillus anthracis*	55
P37949	*Bacillus subtilis*	56
Q65H50	*Bacillus licheniformis*	56
A6QHC7	*Staphylococcus aureus*	54
C1CKU6	*Streptococcus pneumoniae*	54
Q5M4M2	*Streptococcus thermophilus*	56
Q831Z0	*Enterococcus faecalis*	56
Q03QU8	*Lactobacillus brevis*	53
Q03FQ4	*Pediococcus pentosaceus*	53
Q14NN1	*Spiroplasma citri*	53
A7FXL9	*Clostridium botulinum*	55
A6L744	*Bacteroides vulgatus*	53
A6LC18	*Parabacteroides distasonis*	52
Q2S5I1	*Salinibacter ruber*	53
A5U598	*Mycobacterium tuberculosis*	52
I7G5I5	*Mycobacterium smegmatis*	48
Q82BZ3	*Streptomyces avermitilis*	48
Q9Z8I4	*Chlamydia pneumoniae*	50
Q823H7	*Chlaymydophila caviae*	49
Q9PKX6	*Chlamydia muridarum*	49
P60788	*Shigella flexneri*	100
P0A1W5	*Salmonella typhi*	96
A4TKY0	*Yersinia pestis*	92
Q6LMS0	*Photobacterium profundum*	83
Q3IDL4	*Pseudoalteromonas haloplanktis*	75
Q0VP16	*Alcanivorax borkumensis*	74
A9KF98	*Coxiella burnetii*	71
Q87C09	*Xylella fastidiosa*	67
A9III9	*Bordetella petrii*	66
B9J716	*Agrobacterium radiobacter*	57
B9JYH0	*Agrobacterium vitis*	55
Q8UIQ2	*Agrobacterium tumefaciens*	57
Q6G1F5	*Bartonella quintana*	56
Q0C5X0	*Hyphomonas neptunium*	56
Q5NLP5	*Zymomonas mobilis*	59
Q5FHQ1	*Ehrlichia ruminantium*	51
A8F140	*Rickettsia massiliae*	56
C4XND4	*Desulfovibrio magneticus*	60

Multiple Sequence Alignment of the C-terminal domain of EF4/LepA. Black indicates 100%, dark grey 80 - 100%, light grey 60 - 80% and white < 60% conservation. The start of the *E. coli* CTD is indicated (red arrow).

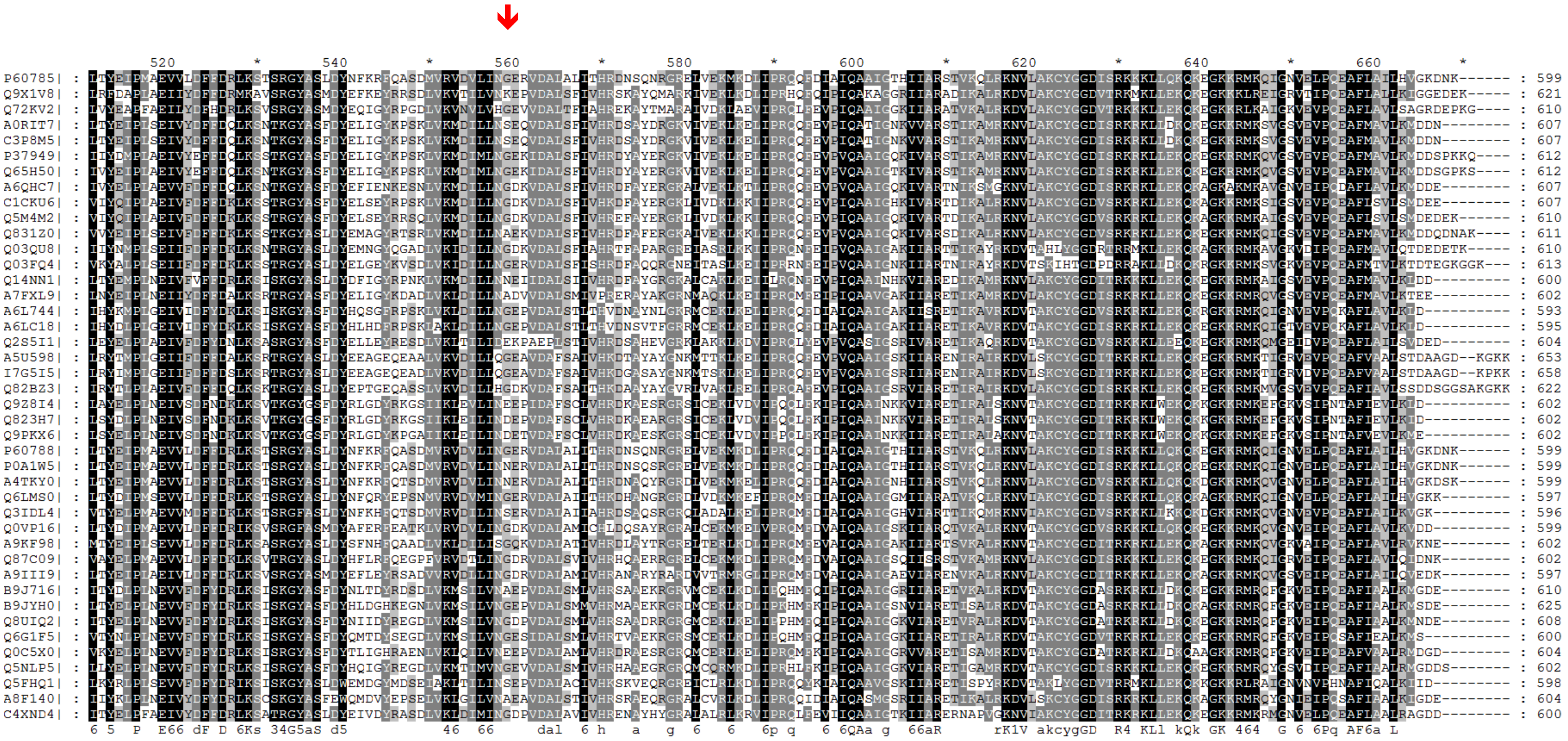

References

1 Laurberg, M. *et al.* Structure of a mutant EF-G reveals domain III and possibly the fusidic acid binding site. *J. Mol. Biol.* **303,** 593-603, doi:10.1006/jmbi.2000.4168 (2000).

2 Evans, R. N., Blaha, G., Bailey, S. & Steitz, T. A. The structure of LepA, the ribosomal back translocase. *Proc. Natl. Acad. Sci. USA* **105,** 4673-4678, doi:10.1073/pnas.0801308105 (2008).

3 Gagnon, M. G., Lin, J., Bulkley, D. & Steitz, T. A. Crystal structure of elongation factor 4 bound to a clockwise ratcheted ribosome. *Science* **345,** 684-687 (2014).
